# A model for skin cancer using combination of ensemble learning and deep learning

**DOI:** 10.1371/journal.pone.0301275

**Published:** 2024-05-31

**Authors:** Mehdi Hosseinzadeh, Dildar Hussain, Firas Muhammad Zeki Mahmood, Farhan A. Alenizi, Amirhossein Noroozi Varzeghani, Parvaneh Asghari, Aso Darwesh, Mazhar Hussain Malik, Sang-Woong Lee

**Affiliations:** 1 Institute of Research and Development, Duy Tan University, Da Nang, Vietnam; 2 School of Medicine and Pharmacy, Duy Tan University, Da Nang, Vietnam; 3 Department of AI and Data Science, Sejong University, Seoul, Republic of Korea; 4 Department of Communication and Computer Engineering, Cihan University-Erbil, Kurdistan Region, Iraq; 5 Electrical Engineering Department, College of engineering, Prince Sattam Bin Abdulaziz University, Al-Kharj, Saudi Arabia; 6 Department of Computer Engineering, Central Tehran Branch, Islamic Azad University, Tehran, Iran; 7 Department of Information Technology, University of Human Development, Sulaymaniyah, Kurdistan region of Iraq; 8 School of Computer Science and Creative Technologies College of Arts, Technology and Environment (CATE) University of the West of England Frenchay Campus, Coldharbour Lane Bristol, Bristol, United Kingdom; 9 Pattern Recognition and Machine Learning Lab, Gachon University, Seongnamdaero, Sujeonggu, Seongnam, Republic of Korea; Sunway University, MALAYSIA

## Abstract

Skin cancer has a significant impact on the lives of many individuals annually and is recognized as the most prevalent type of cancer. In the United States, an estimated annual incidence of approximately 3.5 million people receiving a diagnosis of skin cancer underscores its widespread prevalence. Furthermore, the prognosis for individuals afflicted with advancing stages of skin cancer experiences a substantial decline in survival rates. This paper is dedicated to aiding healthcare experts in distinguishing between benign and malignant skin cancer cases by employing a range of machine learning and deep learning techniques and different feature extractors and feature selectors to enhance the evaluation metrics. In this paper, different transfer learning models are employed as feature extractors, and to enhance the evaluation metrics, a feature selection layer is designed, which includes diverse techniques such as Univariate, Mutual Information, ANOVA, PCA, XGB, Lasso, Random Forest, and Variance. Among transfer models, DenseNet-201 was selected as the primary feature extractor to identify features from data. Subsequently, the Lasso method was applied for feature selection, utilizing diverse machine learning approaches such as MLP, XGB, RF, and NB. To optimize accuracy and precision, ensemble methods were employed to identify and enhance the best-performing models. The study provides accuracy and sensitivity rates of 87.72% and 92.15%, respectively.

## Introduction

The unrestricted growth of skin cells, commonly triggered by exposure to UV radiation, genetic factors, and environmental influences, is a common cause of skin cancer [1]. The sickness involves numerous histological subtypes, with basal cell carcinoma (BCC), squamous cell carcinoma (SCC), and melanoma being the three key types [2]. Basal cell carcinoma, the most prevalent type, generally presents itself as slow-growing lesions on sun-exposed areas, which are characterized by a pearly appearance and rolled edges. Scaly or crusted lesions are a common sign of squamous cell carcinoma, which can metastasize if not identified and treated early, in contrast to other types of cancer.

Despite its high potential for metastasis, melanoma is not a primary concern compared to other cases. It arises from melanocytes, the cells that produce pigment in the skin, and it is characterized by its tendency to display various morphological characteristics and irregular borders. Skin cancer stands as the prevailing and most frequently encountered form of malignancy globally [3]. Annually, there are more than 3.5 million incidences of Melanoma, Basal Cell Carcinoma, and Squamous Cell Carcinoma diagnosed, surpassing the collective occurrences of breast cancer, lung cancer, and colon cancers. Remarkably, Melanoma alone claims a new victim every 57 seconds [4].

Skin cancer detection benefits from using machine learning and deep learning due to their capacity for automated, precise, and effective analysis of skin lesions and images. They facilitate early detection, ensure consistent performance, enable data analysis, and assist in tailoring medical decisions to individuals. Furthermore, these technologies play a role in screening, prioritization, and ongoing improvement, making them well-suited for widespread implementation. Their adoption contributes to the reduction of unnecessary diagnoses and the timely identification of potential skin cancer cases. Due to the importance of proper and accurate diagnosis of the type of skin cancer, this study employed various techniques in machine learning and deep learning to enhance the precision and accuracy of distinguishing between benign and malignant cases of skin cancer.

The contributions in this study are as follows:

- Employing a spectrum of transfer learning models, such as DenseNet-201, DenseNet-121, ResNet-50, ResNet-101, ResNet-152, VGG19, and EfficientNet-B3
- Due to the high number of features after extraction, a range of feature selectors are deployed, namely, ANOVA, XGB, Lasso, PCA, Random Forest, Mutual Information, Univariate, and Variance
- Improving evaluation metrics by deploying different Ensemble techniques and enhancing the quality of the results

The rest of this paper is outlined as follows: Section 2 delves into the discussion of related works; Section 3 explains the suggested approach; Section 4 presents experimental results and discussion; in Section 5, a comparison between others’ work and this paper is depicted; and lastly, section 6 encapsulates the conclusions drawn from the study.

## Related work

Recently, there has been a myriad of inquiries conducted on the matter of skin cancer. At present, multiple research initiatives are progressing toward investigating how machine learning and deep learning methodologies can be added to this field. The healthcare field has seen a substantial surge in interest in computer-assisted diagnosis (CAD), which is a vital area of investigation. This sector encompasses a plethora of research that is currently being scrutinized in the present paper.

Kumar K et al. [5] developed an advanced skin cancer classification and prediction technique using augmented intelligence with the ResNet50 model on Kaggle datasets. Using the Augmented Deep Neural Networking (AuDNN) method, they extracted critical features, identified cancer regions, and improved dataset accuracy through clustering and attribute dependency mapping. This approach achieved 93.26% accuracy in skin cancer classification. Balaha and Hassan [6] developed an automated approach using the Sparrow Search Algorithm (SpaSA) to detect, classify, and segment skin cancer. They used U-Net models and a meta-heuristic optimizer along with pre-trained CNN models on datasets. Tabrizchi et al. [7] devised an automated skin cancer detection model using dermoscopic images and an enhanced VGG-16 CNN architecture. Adla et al. [8] crafted a robust medical decision support system by implementing an optimized full-resolution convolutional network for categorizing skin lesions from dermoscopy images. They introduced a method that fine-tuned hyperparameters using a dynamic graph cut algorithm, effectively addressing segmentation challenges and refining the precision of skin cancer diagnosis. Their model achieved 97.986% accuracy in precisely categorizing skin lesions. Mridha et al. [9] developed deep learning models for skin cancer classification, addressing class imbalance issues. Using the HAM10000 dataset, they trained a CNN, achieving an 82% classification accuracy and 0.47% loss accuracy for seven types of skin cancer. Additionally, an explainable AI system using Grad-CAM and Grad-CAM++. Wu et al [10] introduced a skin cancer classification method using discrete wavelet down-sampling for feature reconstruction, addressing issues of information loss during down-sampling. Their approach integrated a multichannel attention mechanism improving pathological feature utilization. The proposed model achieved 95.84% accuracy. Qasim Gilani et al. [11] used a neural network approach using the surrogate gradient descent method to classify over 6,000 skin lesion images. Their proposed VGG-13 model achieved 89.57% accuracy and a 90.07% F1 score. Huang et al. [12] investigated using hyperspectral imaging (HSI) to detect skin cancer lesions, utilizing the ISIC dataset to train and test models for basal cell carcinoma (BCC), squamous cell carcinoma (SCC), and seborrheic keratosis (SK). They applied the YOLO version 5 to train the model and compared the performance of HSI and RGB classification models. The study revealed that the HSI model showed a 7.5% increase in overall recall rate (0.722 to 0.794) compared to the RGB model, particularly in capturing SCC features, indicating the potential for HSI to improve skin cancer detection. Teodoro et al. [13] addressed the challenges in early diagnosis of skin cancer due to the similarity of symptoms with other diseases, leading to errors in diagnosis. They proposed an approach using EfficientAttentionNet, a CNN architecture, for early detection of melanoma and non-melanoma skin lesions. Their methodology involved several stages: pre-processing skin images, using GAN to balance sample numbers, creating masks for regions of interest via a U-net model, and training EfficientAttentionNet with a mask-based attention mechanism. S M et al. [14] developed a Deep Convolutional Neural Network (DCNN) to classify skin cancer types from dermoscopic images. Using ISIC-2019 and ISIC-2020 datasets, they addressed image resolution differences and class imbalances through augmentation and metadata use. Leveraging the EfficientNet architecture with transfer learning and the ranger optimizer, they achieved superior skin lesion classification results, obtaining an AUC-ROC score of 0.9681 after fine-tuning EfficientNet-B6. The paper [15] introduced deep transfer learning for early skin cancer detection, leveraging pre-trained convolutional neural network models. It employed data augmentation techniques to enhance model robustness and prevent overfitting on MODE-NODE and ISIC skin lesion datasets. Through empirical analysis, vgg19 was identified as the most suitable model, achieving a testing accuracy of 98.8%. Comparative results with existing methods showed vgg19’s superiority in accuracy and efficiency, trained on significantly fewer images. These findings highlighted the efficacy of data augmentation in improving detection while reducing resource consumption. Khan et al. [16] presented a fully automated computer-aided diagnosis (CAD) system for detecting malignant melanoma, a deadly form of skin cancer. It utilized a deep learning framework, including pre-processing, lesion segmentation with MASK-RCNN, and feature extraction with DenseNet. The system achieved accuracy on validation datasets ISBI2016, ISBI2017, and HAM10000. MASK-RCNN also achieved high accuracy on ISBI2016 and ISBI2017. The paper provided a comparison with other state-of-the-art methods, demonstrating the effectiveness of the proposed framework. [17] developed a method for skin cancer detection and recognition using a combination of deep learning and an iteration-controlled Newton-Raphson (IcNR) based feature selection method. It followed three primary steps: lesion localization using Faster R-CNN with a contrast stretching approach based on the bee colony method (ABC), deep feature extraction using DenseNet201, and feature selection with IcNR. The selected features were then used for classification with multilayered feed-forward neural networks. Testing on ISBI2016 and ISBI2017 datasets achieved accuracies of 94.5% and 93.4%, respectively. Results demonstrated the proposed technique’s superior accuracy and efficiency compared to existing methods. Khan et al. [18] proposed a novel deep-learning framework for lesion segmentation and classification. Mask R-CNN was implemented for segmentation, utilizing Resnet50 and feature pyramid network (FPN) as a backbone. Fully connected layers generated final masks. For classification, a 24-layered convolutional neural network was employed, activating based on visualized higher features. The method was validated on PH2, ISBI2016, and ISIC2017 datasets for segmentation and HAM10000 for classification, demonstrating superior performance with high sensitivity (85.57%), precision (87.01%), F1-Score (86.28%), and accuracy (86.5%) compared to existing techniques. To have better overview of all, we put them in Table 1.

**Table 1:** Detailed summaries on predicting and classifying skin cancer in other studies.

References	Dataset	Method
Kumar K et al. [5]	Kaggle+ CIA datasets	AuDNN+ IOT
Balaha and Hassan [6]	ISIC (2016-2017-2018)	Transfer learning and sparrow search algorithm
Tabrizchi et al. [7]	SIIM-ISIC	VGG
Adla et al. [8]	ISIC 2019+ ISIC 2020	FrCN-DGCA
Mridha et al. [9]	HAM10000	Optimized Convolutional Neural Network
Wu et al. [10]	HAM10000	Standard convolution+ wavelet down-sampling
Qasim Gilani et al. [11]	ISIC 2019	Deep Spiking Neural Network
Huang et al. [12]	ISIC Library	YOLOv5
Teodoro et al. [13]	ISDIS	GAN+ RoI-Based
S M et al. [14]	ISIC-2020	DCNN
Mawgoud et al. [15]	MODE-NODE, ISIC	Deep Transfer Learning
Khan et al. [16]	ISBI2016, ISIC2017, HAM10000	Deep learning
Khan et al. [17]	ISBI2016, ISBI2017	Deep Learning with IcNR-based feature selection
Khan et al. [18]	PH2, ISBI2016, ISIC2017, HAM10000	Mask R-CNN, CNN

## Proposed methods

**Fig 1:**
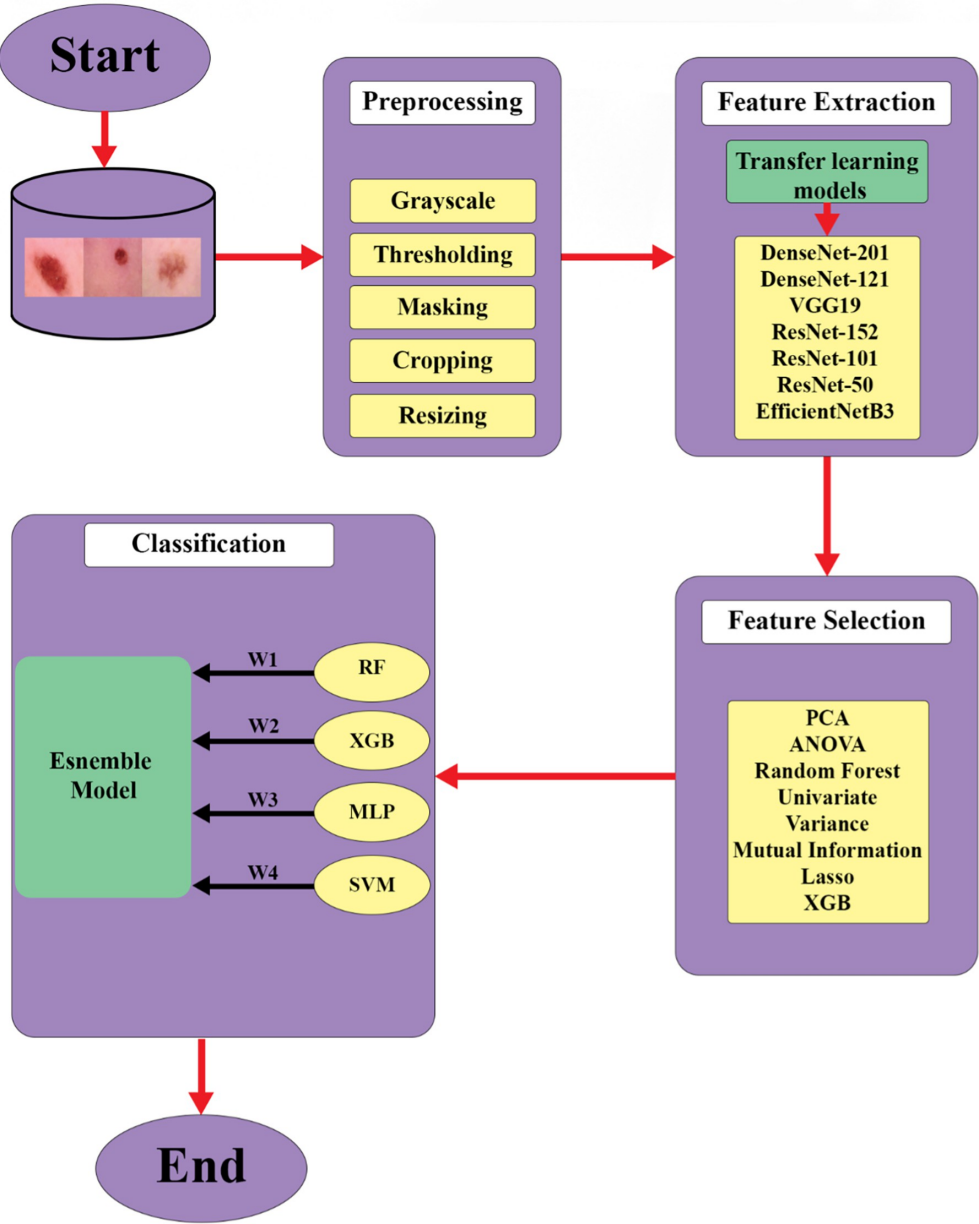
The workflow of the proposed method.

This section provides a comprehensive elucidation of the research approach employed for the diagnosis of skin cancer, encompassing all associated elements. It contains the preprocessing phase, delineates the techniques utilized for feature extraction, and discusses the algorithms applied. A condensed overview of these employed methods is visually represented in Fig 1.

A summary of the applied method is illustrated in Fig 1. It is conceivable that to have better quality images, a pre-processing method was employed. After obtaining new images, a feature extractor was applied to have better data quality and the best and most important features, a feature selector method, namely, Lasso, was employed. Finally, an ensemble method was utilized to increase evaluation metrics.

The novelty in this paper lies in the comprehensive and systematic exploration and combination of these various methodologies, suggesting an in-depth approach to feature extraction, feature selection, and model combination to boost the overall performance.

This research uses a dataset [19] that has a harmoniously balanced compilation of images portraying both benign and malignant skin moles. The dataset is structured into two distinct folders, all captured at a resolution of 224x244 pixels. These images vividly depict the contrasting characteristics of the two-mole types, offering a comprehensive visual resource for discerning the nuances between benign and malignant skin anomalies.

### Preprocessing

The preprocessing of dermoscopic images is paramount in developing precise algorithms for automated skin cancer diagnosis [20]. Skin cancer is among the most widespread types of cancer globally [21]. Early and accurate diagnosis is vital for improving prognoses and survival rates. However, the accuracy of diagnosis heavily relies on the expertise of dermatologists who visually assess and analyze skin lesions from dermoscopic images. Automated algorithms seek to enhance diagnostic precision by utilizing computerized analysis of skin images. Nevertheless, original dermoscopic images often contain flaws such as noise, inconsistent lighting, and other irregularities that can adversely affect algorithm performance. Proficient preprocessing of these images is instrumental in standardizing the data and bolstering algorithm dependability.

The primary objective of preprocessing in skin cancer diagnosis is to enhance image quality and prepare the data for in-depth analysis by the diagnostic algorithm. Fundamental preprocessing steps encompass the conversion of color spaces, removal of hair, correction of uneven illumination, enhancement of contrast, and elimination of noise. The shift from RGB color space to alternative color spaces like CIELAB aids in normalizing skin color and lesion patterns [22]. Noises caused by hair-over lesions are addressed through inpainting, isolating the lesions themselves. Uneven illumination and reflections that may obscure lesions are mitigated through illumination correction, ensuring uniform brightness and heightened contrast. Filtering the image is also pivotal for eradicating extraneous artifacts and balancing noise. Employing these preprocessing techniques helps isolate diagnostically significant features of the lesion for the algorithm to analyze.

Following the fundamental preprocessing steps, additional techniques contribute to standardizing and normalizing specific features within the prepared images. Strategies such as histogram equalization bolster contrast and amplify feature visibility. Normalizing color, size, and structure in the preprocessed images empowers the algorithm to characterize, categorize, and compare lesions more accurately. Data augmentation through rotation, scaling, and flipping can amplify dataset size and variability, enhancing algorithm training. Apt preprocessing produces refined, normalized image data, aiding complex algorithms in extracting subtle features and patterns for precise diagnosis.

In essence, meticulous preprocessing of dermoscopic images diminishes noise, and distracting variability while accentuating the visually meaningful diagnostic features of skin lesions. Standardized, improved images facilitate machine learning algorithms in effortlessly identifying patterns indicative of cancerous or benign lesions. By establishing robust preprocessing pipelines tailored to the specific challenges of dermoscopic images, researchers can formulate highly accurate algorithms for automated skin cancer screening and diagnosis. Dependable preprocessing facilitates the seamless real-world integration of these systems, augmenting public access, efficiency, and the quality of skin cancer diagnosis.

In this research, RGB-formatted images transform grayscale. Subsequently, a binary inverse thresholding operation using Otsu’s method is applied. This operation provides a binary mask that accentuates the object of interest against its backdrop [23]. Employing this mask, the object is then segregated from the background within the RGB image through bitwise operations. Pixels representing the background are altered to white, erasing the background itself. Through the identification of indices corresponding to non-zero (foreground) pixels in the processed image, the parameters of the bounding box enclosing the object are discerned. This information facilitates the extraction of a cropped variant of the RGB image, encompassing solely the object. The cropped image is subsequently resized to conform to a standardized dimension of 224x224 pixels, ensuring uniformity in input measurements. Ultimately, the resized image is incorporated into a roster of images designated for subsequent analysis. This meticulous preprocessing sequence culminates in the creation of a collection of images, uniformly sized and centered. These images are poised for subsequent computational tasks, such as image classification or object recognition.

### Feature extraction

Extracting features is a crucial stage in advancing machine learning systems for automated skin cancer diagnosis. While preprocessing of dermoscopic images normalizes and enriches the initial data, feature extraction precisely identifies and segregates the distinct visual attributes and patterns present in the lesions. Thoughtful selection and extraction of only the most diagnostically pertinent features facilitate more effective and focused analysis, concurrently reducing complexity for the classification algorithm. The objective is to derive a set of features that can faithfully represent each image, enabling the system to discern discerning patterns distinguishing between malignant melanomas and harmless lesions.

Features at the pixel level, such as color, texture, and shape, alongside more complex semantic features describing asymmetry, border irregularity, color variations, and dimensions, offer potential value in distinguishing cancerous and non-cancerous skin lesions. The choice of extraction techniques depends on the priority of features in the computer vision approach. For pixel-level features, standard methods involve segmentation, thresholding, edge detection, and filtering to isolate colors, textures, and structures. Segmentation divides the image into regions with comparable traits [24]. Thresholding emphasizes pixel intensities indicative of boundaries and shapes [25]. Edge detection identifies lines and abrupt changes in the image [26]. Gabor filters and other filtering methods characterize texture patterns [27].

In contrast, extracting higher-level diagnostic features necessitates algorithms to quantify semantic attributes and patterns in the lesions. These algorithms can pinpoint and measure asymmetry across multiple axes of the lesion. Algorithms gauging border irregularity evaluate the smoothness versus jaggedness of the lesion edges. Others assessing color variations can classify multiple colors and uniformity of color across the lesion area. Algorithms for dimensions extract size characteristics and dimensional ratios. A meticulous selection of a concise set of the most valuable features for diagnosis prevents unnecessary complexity in the model while enabling it to discern the visual patterns that most consistently set apart malignant and benign skin lesions.

Strategic feature extraction empowers machine learning models to pinpoint and concentrate on the most diagnostically meaningful elements within dermoscopic images. Condensing each image to its essential array of distinguishing visual features augments efficiency and precision while minimizing model intricacies. Coupled with robust preprocessing, deliberate feature extraction provides optimized data for algorithm training and evaluation in automated skin cancer diagnosis. The use of more focused data enhances model performance and facilitates integration into actual clinical settings, thus advancing dermatological care.

In the realm of skin cancer analysis, the integration of pre-trained convolutional neural network (CNN) models as feature extractors has ushered in a transformative approach to enhancing diagnostic accuracy. Amidst the diverse array of models employed for this purpose, such as DenseNet-121 [28], VGG19 [29], ResNet-50, ResNet-101, ResNet-152 [30], and EfficientNet-B3 [31], a rigorous performance evaluation process spotlighted DenseNet-201 as the preeminent contender. These pre-trained CNN models, having undergone comprehensive training on extensive image datasets, exhibit an innate capability to learn and encapsulate hierarchical features from input images autonomously. This intrinsic trait enables them to undertake efficient feature extraction sans the need for labor-intensive manual feature engineering. Of noteworthy significance, DenseNet architectures, characterized by densely interwoven layers, proffer an exclusive advantage by fostering unimpeded information flow among layers bolstered by intricate connectivity patterns. This unique architectural structure equips the models with an exceptional ability to simultaneously capture both intricate details at the lower level and complicated, complex features at the higher level. As a result, these models are highly proficient in detecting subtle nuances present within dermatological images—nuances that often hold critical significance as indicators of potential skin cancer.

### Feature selection

While feature extraction detects promising attributes within images, feature selection takes a step further by narrowing down the data to the most pertinent characteristics crucial for skin cancer diagnosis. Not all the extracted features have valuable insights to distinguish between malignant melanomas and harmless lesions. Feature selection assesses the extracted features and cherry-picks a subset that possesses the optimal discriminating potential for precise classification. This streamlining process enhances the efficiency, accuracy, and comprehensibility of the model by eliminating superfluous, inconsequential, or bewildering features from the dataset.

Efficient feature selection mandates analytical techniques to gauge and rank the utility of the extracted features. Commonly, methods like filters, wrappers, and embedded approaches are employed for this purpose. Filter techniques use statistical metrics like correlation, mutual information, or chi-square tests to score and rank features independently of the model [32]. Wrappers utilize the model itself to test various feature combinations and assess the model’s performance [33]. Embedded methods conduct feature selection as an integral part of the model construction process [34]. In the context of skin cancer diagnosis, a blend of these techniques is often necessary to pinpoint the most compelling features.

Prudent feature selection scrutinizes texture, color, shape, and semantic attributes to ascertain which offers the highest discriminatory potential. The optimal colors distinguishing melanomas from benign lesions are singled out. Specific texture patterns can indicate malignancy. Asymmetry, border irregularity, and dimensional ratios with notable discriminatory capabilities are retained, while redundant features are discarded. This streamlining and reduction of the feature space facilitate more efficient algorithm training, preventing distraction from irrelevant inputs.

By removing redundant and non-informative inputs, feature selection guides the model to concentrate solely on the most relevant components essential for optimal performance. This not only enhances model interpretability but also augments predictive accuracy. The outcome is an optimized subset of features that allows effective training of machine learning models for automated analysis of dermoscopic images, ultimately improving melanoma detection.

Following the phase of extracting features using DenseNet-201 in this study, a crucial step is introduced to enhance and optimize the obtained feature set. In this context, a significant technique known as the Lasso method is introduced for feature selection. Lasso, or Least Absolute Shrinkage and Selection Operator, holds prominence as a sophisticated regularization method that achieves both feature selection and coefficient reduction. Operating within the framework of linear regression, Lasso integrates a penalty term that encourages specific coefficients to be precisely reduced to zero, effectively removing the associated features [35]. The utilization of Lasso in this investigation involves a thorough assessment of the relevance of each extracted feature concerning the classification task. By capitalizing on Lasso’s inherent ability to effectively trim irrelevant or redundant features while retaining the most informative ones, the feature set is refined to encompass only those features that substantially contribute to the given task. This refinement enhances the model’s generalization capability and reduces overfitting risks. The integration of Lasso as a feature selector enhances the overall efficiency and comprehensibility of the classification model. Through this unified approach, the study aims to attain an improved feature representation that not only captures the complexities of dermatological images but also leads to enhanced performance in skin cancer classification.

### Classification

The accurate categorization of skin lesions as either malignant or benign stands as a pivotal aspect in the creation of dependable automated frameworks for skin cancer screening and diagnosis [36]. While ensuring the quality of image pre-processing, meticulous feature extraction and judicious filtering of features help optimize the input data, the onus of making the ultimate diagnostic determination lies with the classification algorithm. Given the global prevalence of skin cancer, including melanoma, enhancing screening and diagnostic precision holds tremendous advantages concerning early detection, treatment, and survival rates. Advanced classification algorithms possess the potential to either equal or potentially surpass the diagnostic capabilities of human dermatologists by discerning subtle patterns within dermoscopic images.

The classification algorithm thoroughly evaluates the refined set of visual features extracted from the pre-processed dermoscopic images and sets decision boundaries to classify new samples as either malignant or benign consistently. A broad array of machine learning classification methodologies can be experimented with and fine-tuned to pinpoint the most effective diagnostic approach for the skin lesion classification task. Commonly employed classification algorithms encompass logistic regression, support vector machines, random forests, artificial neural networks, gradient boosting machines, and diverse ensembles. Logistic regression models gauge probability scores based on the feature inputs [37], indicating the likelihood of malignancy or benignity. Support vector machines ascertain optimal hyperplanes between classes in the multivariate feature space [38]. Random forests construct collections of decision trees based on random subsets of features to amplify the overall performance [39]. Artificial neural networks utilize hidden layers to capture intricate nonlinear relationships between image features and diagnostic outcomes.

Within each algorithm, exhaustive parameter adjustment through grid or random searches identifies the optimal model settings and architecture to maximize classification accuracy. Fine-tuning kernels, regularization terms, tree depths, hidden layers, activation functions, and other parameters offer flexibility to tailor the model’s intricacy to suit the challenge posed by the skin image data. Robust scoring metrics like AUC-ROC, precision, recall, and F1-score through cross-validation prevent overfitting and provide an objective understanding of model generalizability. The most effective classification model can then be solidified by training on the entire refined dataset.

After being rigorously optimized through this stringent process, the classification model can consistently categorize new dermoscopic images with a high degree of accuracy. This paves the way for the development of fully automated skin cancer screening systems that can be clinically validated before responsible real-world implementation. These systems have demonstrated the potential to enhance diagnostic speed, objectivity, accessibility, and accuracy. Prudent and responsible use of AI-based classification has the potential to significantly benefit skin cancer patients through early detection, cost reduction, improved outcomes, and potentially saving lives. Although challenges remain for full integration into clinical practice, advanced machine learning classification algorithms hold immense promise to assist dermatologists and enhance skin cancer diagnosis.

Ensemble techniques, a prominent facet within the realm of machine learning, have garnered considerable attention for their exceptional capacity to enhance predictive accuracy and broaden applicability across diverse domains, including the nuanced domain of medical image analysis. The underlying principle of these methods involves amalgamating multiple individual models to craft a more resilient and precise ensemble model. Particularly in the realm of skin cancer detection, where accuracy and precision hold paramount importance, ensemble methods offer an efficacious avenue to elevate the performance of machine learning algorithms. A range of ensemble methodologies exists, each distinguished by unique attributes contributing to their efficacy. For instance, Bagging (Bootstrap Aggregating), demonstrated through the example of Random Forest, entails constructing numerous decision tree models by training them on distinct subsets of the training data [40]. These models’ predictions are subsequently consolidated to have a definitive outcome, effectively countering overfitting while augmenting robustness. On the other hand, Boosting iteratively trains modest models, such as shallow decision trees, by assigning elevated weights to misclassified instances [41]. This iterative approach culminates in a robust ensemble model. Stacking, a more intricate approach, encompasses training multiple foundational models, subsequently training a meta-model to proficiently amalgamate the predictions of these foundational models [42]. The Random Subspace Method (Feature Bagging) entails training each foundational model on random feature subsets, a strategy that effectively mitigates the risk of overfitting [43]. In the context of skin cancer detection, ensemble techniques hold considerable promise, primarily attributable to the intricacies and diverseness inherent in dermatological images. The task of distinguishing between benign and malignant lesions necessitates capturing intricate patterns that a solitary algorithm might struggle to encapsulate fully. Here, ensemble methods emerge as a viable solution by amalgamating the strengths of multiple algorithms, thus countering individual shortcomings and augmenting overall accuracy. In the research endeavor, an ensemble comprising diverse machine-learning algorithms was harnessed for skin cancer detection. This ensemble encompassed XGBoost, renowned for its gradient-boosting mechanism that iteratively hones the model’s predictions, giving precedence to misclassified instances [44]. MLP (Multi-Layer Perceptron), a neural network architecture with multiple concealed layers capable of capturing intricate data relationships, contributed to its distinctive prowess [45]. The MLP architecture utilized in this study embodies a feedforward neural network structure, comprising distinct layers tailored for the effective classification of skin cancer lesions. The input layer encapsulates the flattened representation of extracted image features, aligning with the dimensions of the resized and preprocessed skin lesion images. Throughout the architecture, multiple hidden layers, characterized by varying neuron counts, facilitate the learning of intricate patterns inherent in the input data. Employing ReLU activation functions within these hidden layers ensures efficient gradient propagation and fosters rapid convergence during model training. Additionally, the output layer is equipped with a sigmoid activation function, facilitating the generation of probabilistic outputs suitable for binary classification tasks. Alongside these core components, the MLP architecture integrates dropout layers to mitigate overfitting, batch normalization techniques to stabilize training dynamics, and regularization mechanisms to enhance model generalization. These architectural nuances collectively empower the MLP to discern nuanced features and achieve robust classification performance, thus rendering it a pivotal asset in the realm of skin cancer detection through medical imaging. The ensemble further incorporated SVM (Support Vector Machine), celebrated for its proficiency in segregating data [46] into distinct classes by defining a hyperplane, and Random Forest, an assemblage of decision trees acclaimed for their robust generalization capacity [39]. Each of these algorithms contributed unique strengths to the ensemble’s arsenal. The ensemble amalgamation of XGBoost, MLP, SVM, and Random Forest within this research exemplifies the potential of such a collaborative approach. By synergistically harnessing the individual strengths of these algorithms, the ensemble aimed to attain elevated precision and robustness in detecting malignant lesions. This collaborative strategy is particularly powerful in incorporating a diverse array of features, thereby augmenting the model’s efficacy in discerning between benign and malignant cases. In a rapidly evolving landscape of machine learning, ensemble methods stand as a steadfast route to expanding the horizons of medical image analysis and elevating patient care.

## Discussion and results

In this section, a detailed analysis of the middle steps, process, and performance of the proposed technique for skin cancer detection using machine learning and deep learning methods is presented. Beginning with an examination of each stage’s intricacies, followed by a comprehensive evaluation of the performance metrics and a comparison with alternative methods.

The outcomes of employing various pre-trained transfer learning models and distinct classification algorithms were elucidated. The techniques outlined in this paper are reproduced in a Google Colab, making use of the Python programming language.

The proposed technique encompasses several key stages, each critical for achieving accurate and reliable skin cancer detection. Image preprocessing involves advanced techniques to enhance image quality and remove noise, addressing common challenges such as illumination variations and artifacts inherent in dermatological images. Specifically, Otsu’s method was employed to ensure high-quality data for subsequent processing.

Feature extraction utilized pre-trained deep learning models such as DenseNet-201 to capture discriminative features from dermoscopic images. Additionally, the Lasso method was employed as a feature selector to streamline the feature space and maximize the discriminative potential of the extracted features.

The classification stage involved the application of diverse algorithms, including XGBoost, Multi-Layer Perceptron (MLP), Support Vector Machine (SVM), and Random Forest. Each algorithm was fine-tuned and optimized to delineate between malignant and benign skin lesions, with careful consideration given to parameter tuning and model architecture.

Rigorous evaluation of the proposed technique assessed its performance in skin cancer detection using standard evaluation metrics such as accuracy, precision, recall, and F1-score. A comparative analysis with alternative methods showcased the superior performance of the technique relative to existing approaches. DenseNet-201 emerged as the optimal feature extractor, outperforming other pre-trained models such as ResNet and VGG19.

The evaluation of this research is predicated upon its classification performance, gauged by different evaluation metrics. This study uses accuracy, precision, recall, and F1-score to assess the performance of the proposed method. All the performance metrics are computed as shown in Table 2.

**Table 2:** The performance metrics.

Parameter	Value
Accuracy	$\frac{True\;Positive+True\;Negative}{True\;Positive+True\;Negative+False\;Positive+False\;Negative}$
Precision	$\frac{True\;Positive}{True\;Positive+False\;Positive}$
Recall	$\frac{True\;Positive}{True\;Positive+False\;Negative}$
F1-Score	$2\cdot \frac{Precision\times \;Recall\;}{Precision+Recall\;}$

**Fig 2:**
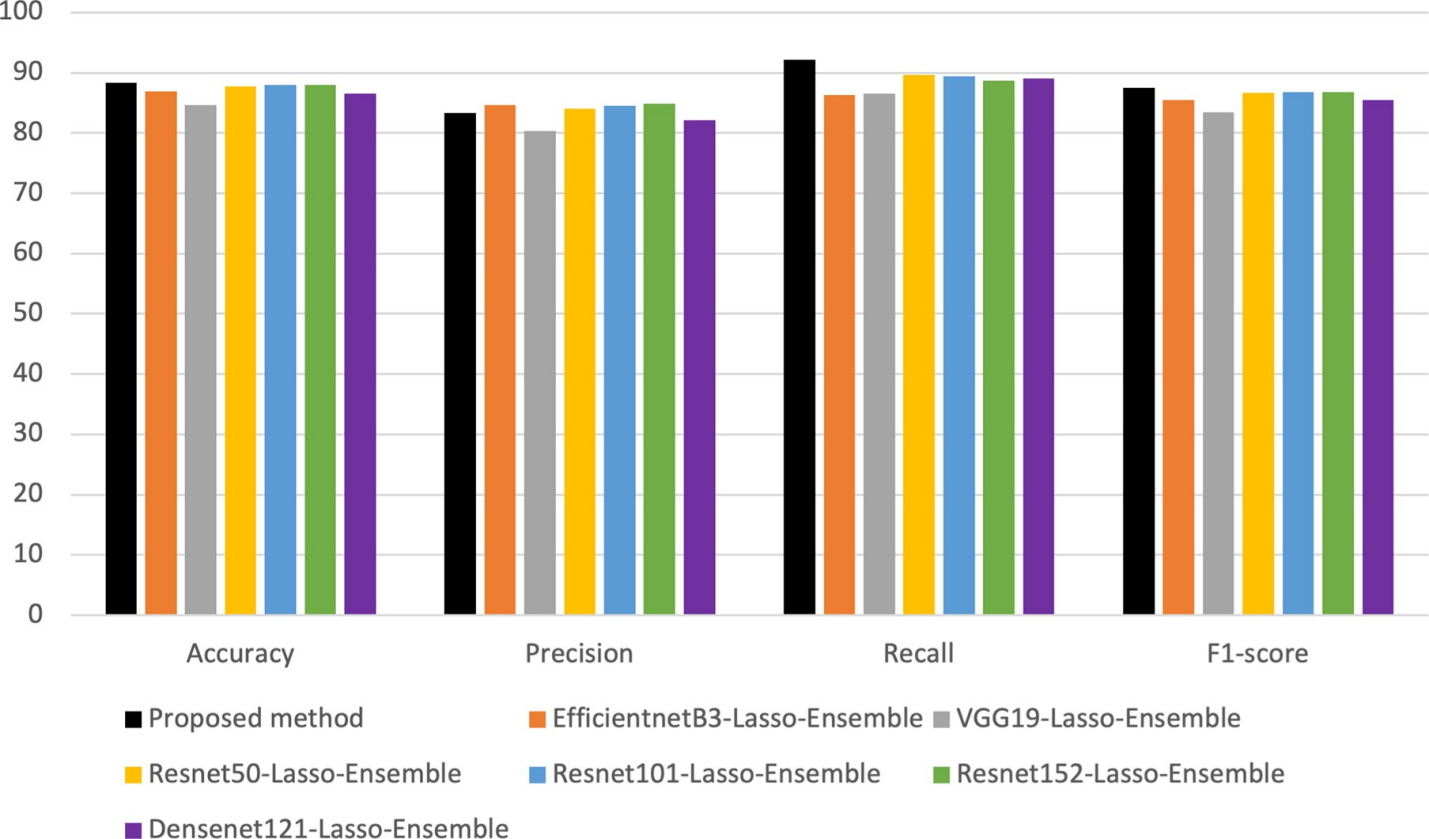
Comparison of different feature extractors.

Following a comprehensive comparative analysis, the superiority of DenseNet-201 consistently emerged. The model’s capability to comprehend and encapsulate finely detailed attributes played a pivotal role in significantly enhancing precision when distinguishing between malignant and benign skin lesions. It is this exceptional aptitude for capturing intricate aspects that firmly establish the prominence of DenseNet-201 as the optimal choice among its counterparts. Fig 2. illustrates a comparative evaluation of DenseNet-201 concerning other pre-trained models employed in the study.

Based on the observations derived from Fig 2., it can be deduced that the outcomes obtained through the proposed methodology exhibit superior performance when compared to alternative techniques, specifically the Resnet family, EfficientnetB3, and VGG19.

After finding DenseNet-201 as the best feature extractor, Lasso, as a feature selector, provides a streamlined and optimized subset of features that maximizes the distinguishing potential of the extracted features. In this study, an extensive ensemble strategy was implemented to enhance the accuracy of skin cancer detection. The ensemble involved the integration of various machine learning models, encompassing XGBoost (XGB), Multi-Layer Perceptron (MLP), Support Vector Machine (SVM), and Random Forest. The stacking technique was applied for the ensemble process, merging predictions from individual models to provide a conclusive decision.

**Fig 3:**
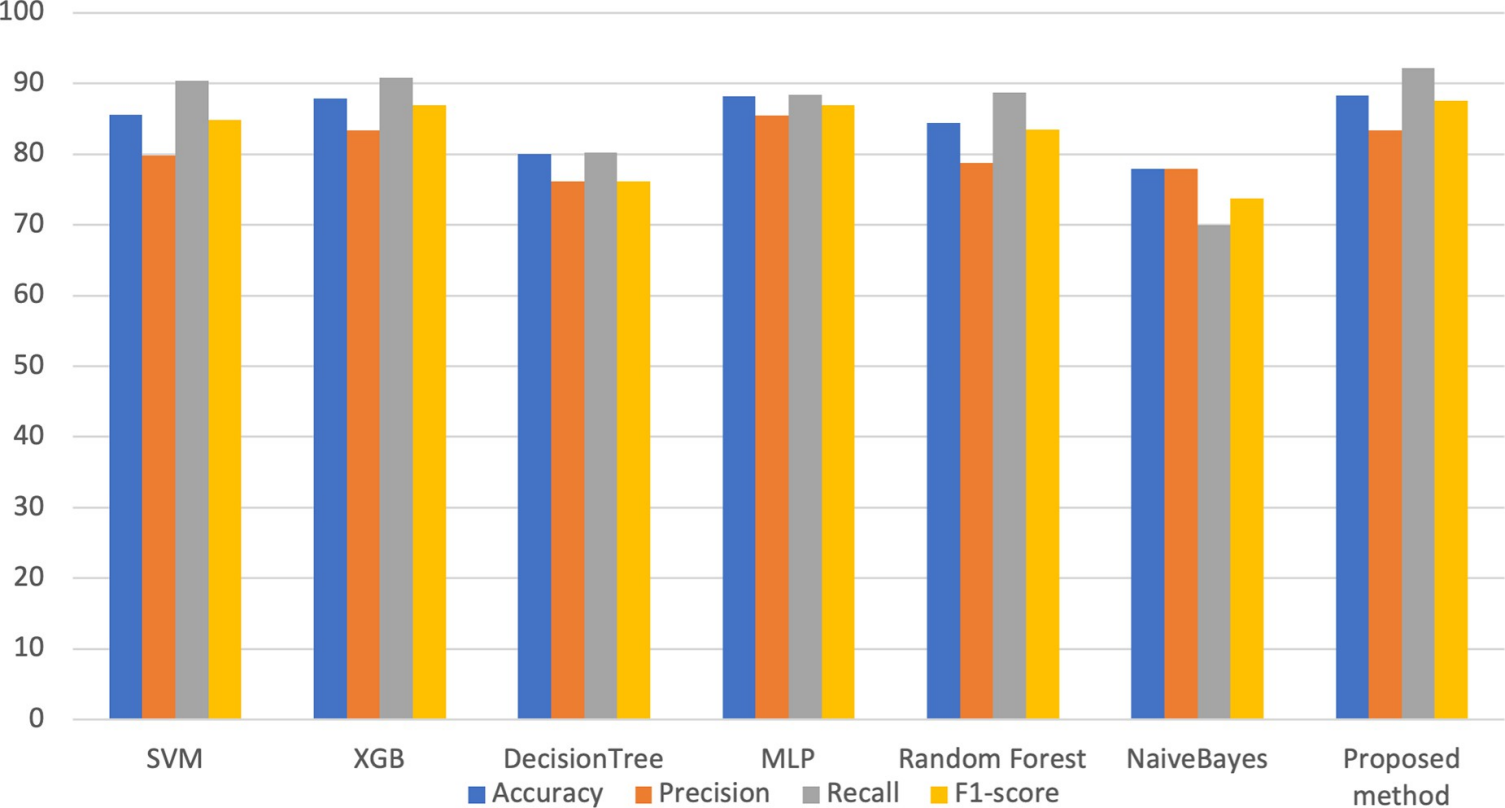
Obtained metrics in the proposed method and other applied classifiers.

Fig 3. provides a visual representation of the metrics’ improvement resulting from implementing the proposed methodology.

The ensemble strategy, incorporating diverse machine learning models, further enhanced the accuracy and robustness of the approach. Fig 3. reveals that the proposed method excels particularly in the recall metric, which is among the most important in the healthcare context [47]. More specifically, the achieved recall rate of 92.15% surpasses that of alternative methodologies, while the attained accuracy rate of 87.72% also demonstrates superior performance relative to competing approaches.

**Fig 4:**
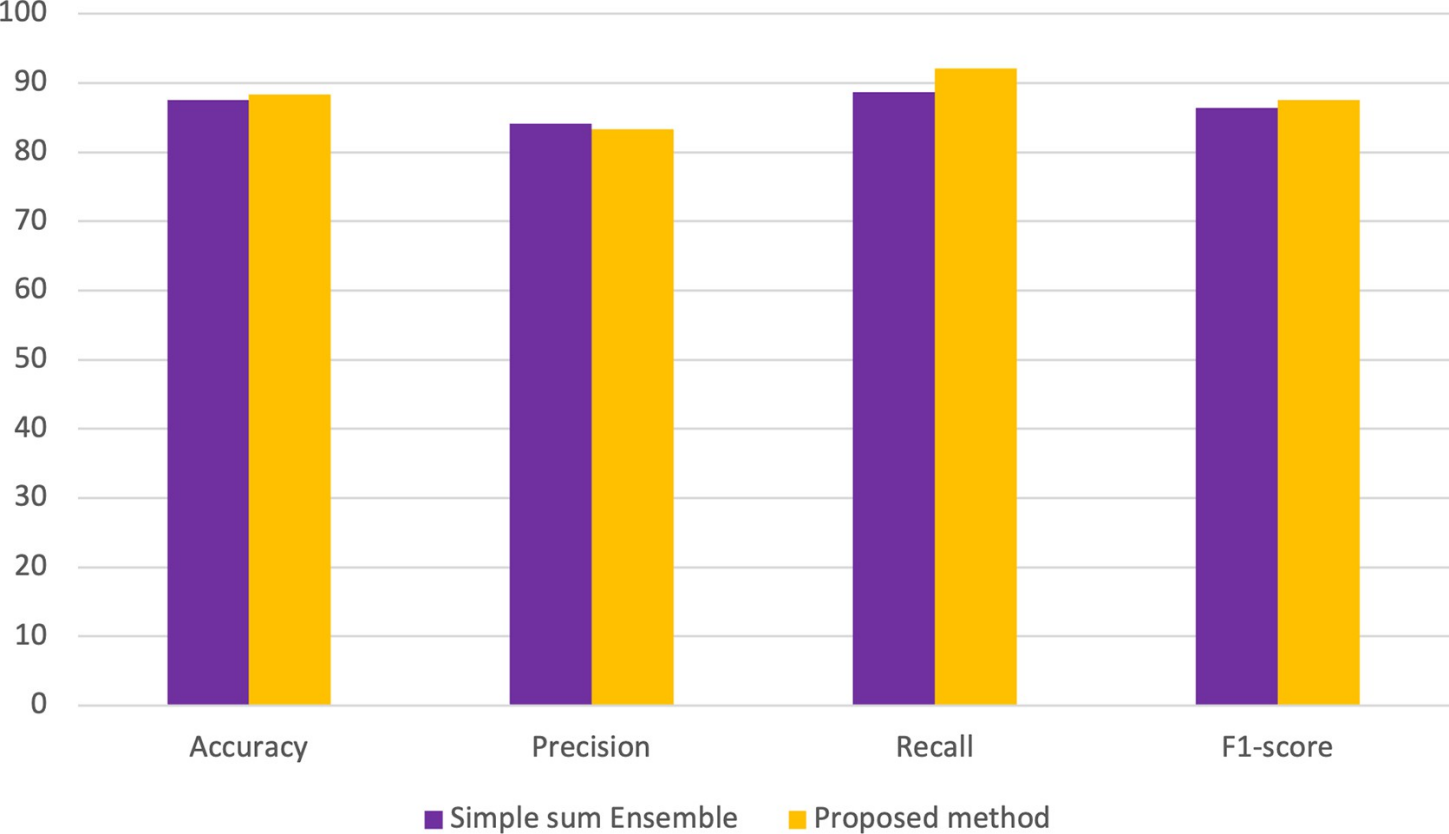
A comparative analysis of the achieved outcomes derived from the proposed methodology in comparison with a straightforward ensemble approach.

Two types of ensembles, whose results are shown in Fig 4., were applied, in which the proposed method was chosen because of its potential.

Fig 4. is a comprehensive comparative analysis to assess the performance outcomes by the novel methodology in contrast to a basic ensemble approach. Findings indicate that the proposed method outperforms the simple ensemble method in three out of four evaluation metrics.

**Fig 5:**
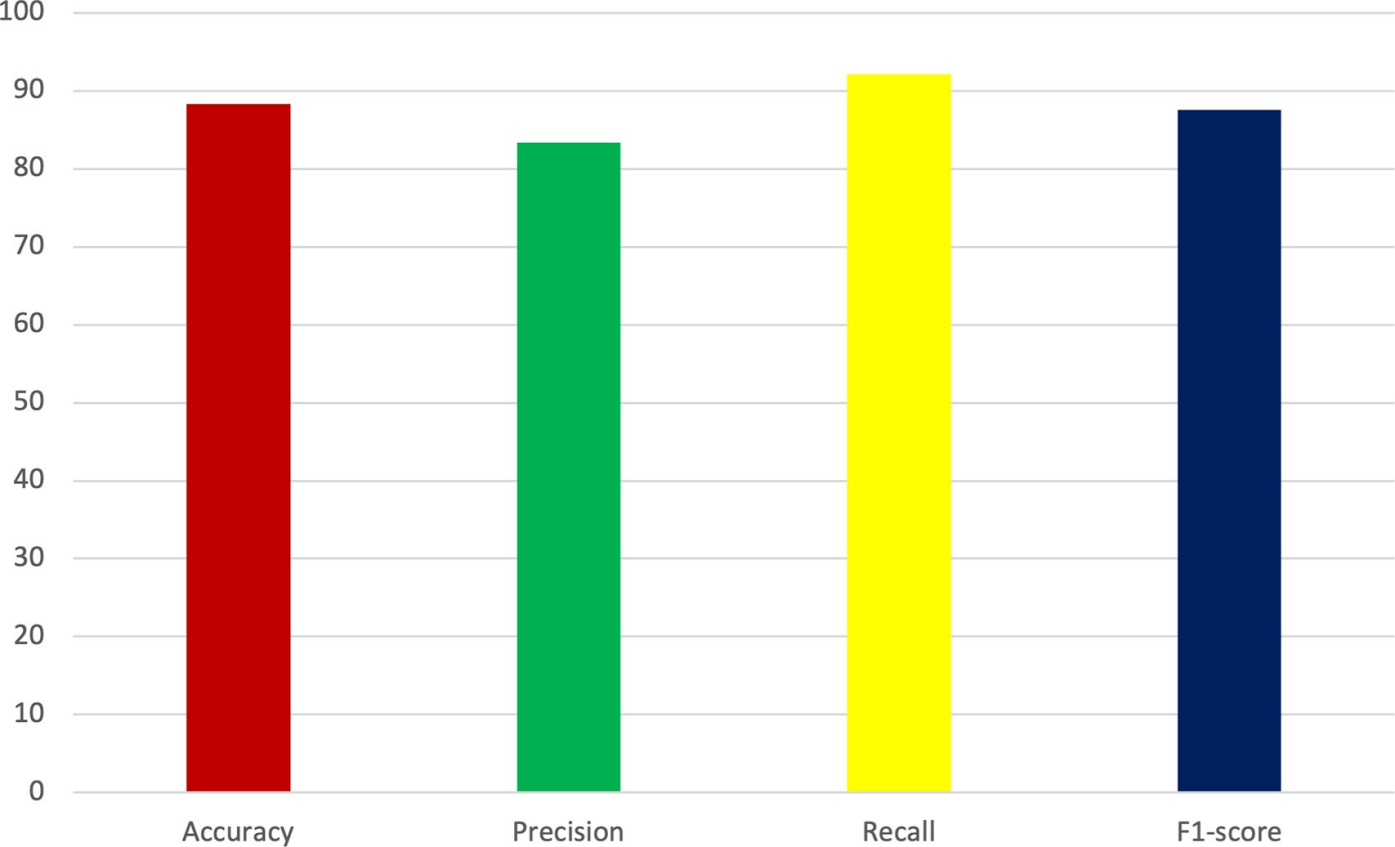
Obtained metrics in the proposed method and other applied classifiers.

Notably, the importance of each model was acknowledged through the allocation of weights proportionate to their respective accuracies. Notably, the MLP model, exhibiting the highest accuracy within the ensemble, received the greatest weight. Subsequently, XGBoost, while offering accuracy slightly below MLP but surpassing SVM and Random Forest, was assigned the second-highest weight. SVM, ranking third in terms of accuracy, followed by Random Forest, with the fourth-highest accuracy, received progressively reduced weights within the ensemble framework. The stacking ensemble technique effectively harnesses the collective capabilities of diverse machine learning models. The evaluation metrics acquired are depicted in Fig 5.

As illustrated in Fig 5., the metrics obtained in the study demonstrate promising applicability within the realm of healthcare. By strategically determining weights based on accuracy, the stacking approach optimally exploits the distinct strengths of individual models, enhancing the final decision-making process. This ensemble method emphasizes a tactical fusion of models, wherein their cooperative outcomes surpass the performance of unique models. This convergence results in a more robust and refined diagnostic system, amplifying its effectiveness in detecting skin cancer.

**Fig 6:**
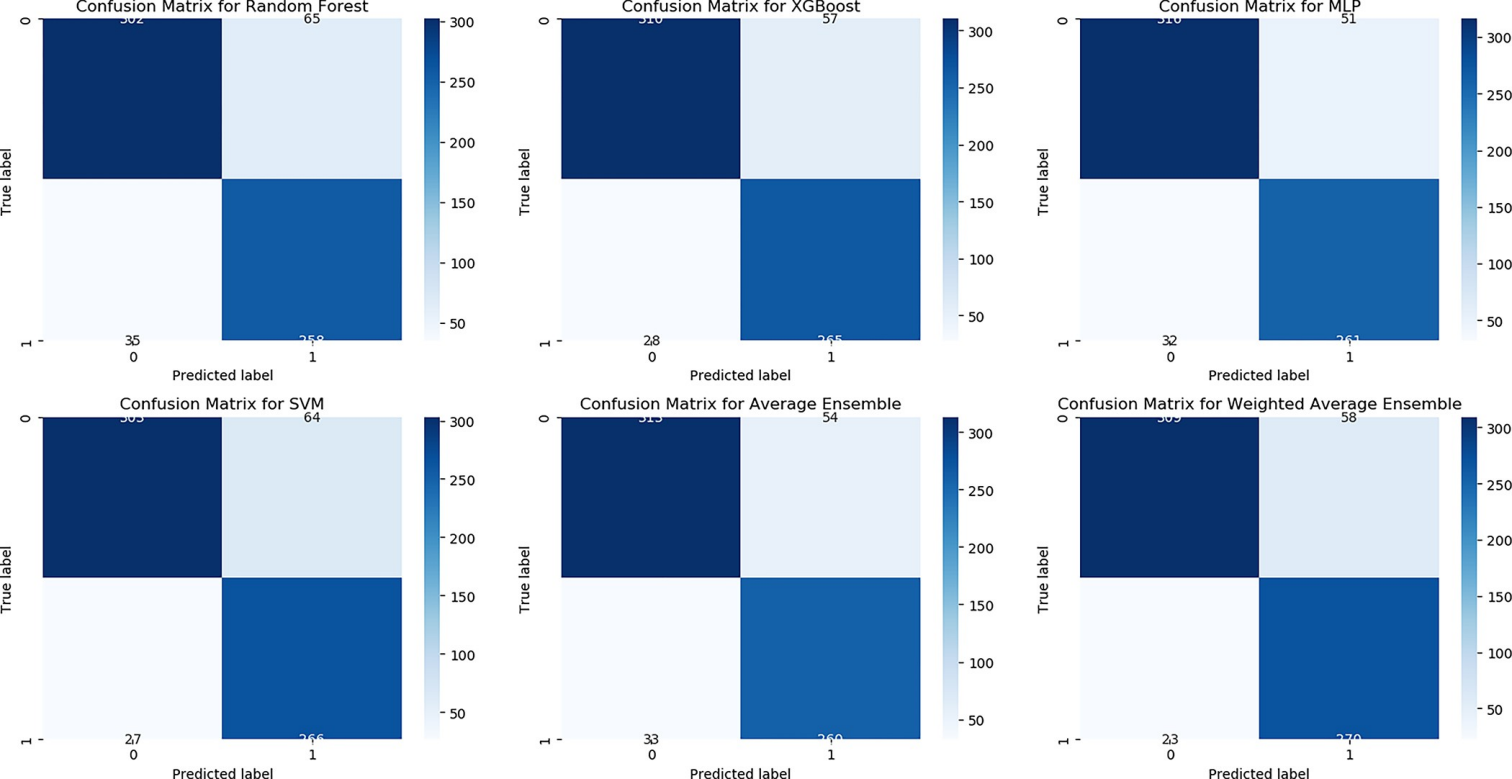
Confusion matrix in used classifiers.

A confusion matrix is a performance measurement tool for classification problems, displaying the number of true positive (TP), true negative (TN), false positive (FP), and false negative (FN) predictions made by a classification model. It provides a comprehensive overview of a model’s accuracy, precision, recall, and F1-score, facilitating performance evaluation, model comparison, error analysis, threshold selection, and class imbalance handling. By analyzing TP, TN, FP, and FN rates, identify common prediction errors, optimize decision thresholds, and mitigate bias in datasets with class imbalance, enhancing the credibility of research findings in the field of machine learning. In Fig 6. the confusion matrix achieved in this paper is visualized.

The ensemble methodology adopted in this study holds significant potential for advancing medical image analysis, particularly within the domain of dermatological imagery. By judiciously amalgamating models with varying strengths and employing stacking as the mechanism of amalgamation, this research seeks to elevate the precision of skin cancer detection. This endeavor, in turn, augments the potential for accurate and timely medical intervention, reflecting a promising stride toward improved patient care.

## Comparison with cutting-edge models

Table 3 presents a comparison of diverse studies employing different methods to classify skin cancer along with their respective outcomes. Mijwil [48] utilized InceptionV3 CNN to classify skin cancer images with 86.90% accuracy, demonstrating its effectiveness in distinguishing benign and malignant cases. Jasil and Ulagamuthalvi [49] explored pre-trained models like VGG16, VGG19, and Inception V3, achieving 74–77% accuracy in classifying skin lesions and highlighting their potential in medical image processing. Dubal et al. [50] introduced a technique using ordinary cameras and Neural Networks to accurately identify cancerous skin abnormalities through visual segmentation. Brinker et al. [51] focused on the binary categorization of skin abnormalities using dermoscopic images and annotations from specialists. Majtner et al. [52] developed an improved melanoma detection method combining deep learning with LDA for feature reduction and enhanced classification accuracy. Gupta et al. [53] presented a methodology enhancing dynamic training and testing procedures for more pronounced outcomes, emphasizing resource-intensive augmentation strategies. Murugan et al. [54] addressed skin cancer diagnosis, focusing on melanoma, employing watershed segmentation and various classifiers, with SVM proving the most effective. Mustafa et al. [55] introduced a method using color space, luminance, and SVM for categorizing benign/malignant lesions based on shape characteristics, emphasizing the SVM’s capability. Linsangan and Adtoon [56] focused on non-invasive skin cancer detection, achieving 86.67% accuracy using geometric features on a Raspberry Pi. Shalu and Kamboj [57] developed a melanoma detection system using preprocessing and segmentation techniques. With an accuracy of 87.72% and sensitivity of 92.15%, the results of this paper surpass many of the mentioned methodologies, signifying a notable enhancement in the accuracy and sensitivity of skin cancer based on the employed techniques.

**Table 3:** Comparison with other studies.

References	Dataset	Method and Methods Used	Accuracy	Sensitivity
Jasil and Ulagamuthalvi [49]	ISIC	Transfer learning	77	-
Dubal et al. [50]	-	Neural Networks	76.9	-
Brinker et al. [51]	ISBI2016	CNN	83.9	56
Gupta et al. [53]	ISIC2016	Ensemble techniques	87	67
Murugan et al. [54]	ISIC	Random Forest	76.87	78.43
Mustafa et al. [55]	-	SVM	80.00	71.43
Linsangan and Adtoon [56]	ISIC archive	KNN	86.67	-
Shalu and Kamboj [57]	MED-NODE	Decision Tree	82.35	-
Proposed method	ISIC	DenseNet-201+ Lasso+ Ensemble learning	87.72	92.15

## Conclusion

This paper aims to assist medical professionals in accurately distinguishing between benign and malignant skin cancer cases using machine learning and deep learning methods. Among the pre-trained models, including ResNet-50, ResNet-101, ResNet-152, VGG19, EfficientNet-B3, and DenseNet-121, DenseNet-201 has been chosen as the feature extractor. In the subsequent phase, the Lasso method is employed as a feature selector, followed by the implementation of diverse machine learning techniques. The innovation lies within the meticulous and systematic investigation and fusion of diverse methodologies, signifying a profound approach towards extracting features, selecting crucial elements, and amalgamating models. The best accuracy and precision are selected and improved through ensemble methods. Given the importance of the sensitivity metric in disease detection, efforts have been directed toward achieving a substantial value. The accuracy and sensitivity values achieved in this study are 87.72% and 92.15%, respectively. The clinical relevance and potential impact of the proposed technique on dermatological practice are profound. By leveraging state-of-the-art machine learning and deep learning methods, a reliable and efficient solution for early detection and diagnosis of skin cancer is offered. The approach holds promise for reducing healthcare costs. In future research endeavors, further refinement and validation of the technique using diverse datasets and advanced deep-learning architectures are planned. Additionally, exploration of the integration of complementary modalities such as clinical data and histopathological findings to enhance diagnostic accuracy is underway.
